# Stimulation of fracture mineralization by salt-inducible kinase inhibitors

**DOI:** 10.3389/fbioe.2024.1450611

**Published:** 2024-09-16

**Authors:** Kaveh Momenzadeh, Diana Yeritsyan, Mohammadreza Abbasian, Nadim Kheir, Philip Hanna, Jialiang Wang, Pere Dosta, Garyfallia Papaioannou, Sarah Goldfarb, Cheng-Chia Tang, Eliz Amar-Lewis, Michaela Nicole Prado Larrea, Edith Martinez Lozano, Mohamed Yousef, John Wixted, Marc Wein, Natalie Artzi, Ara Nazarian

**Affiliations:** ^1^ Musculoskeletal Translational Innovation Initiative, Beth Israel Deaconess Medical Center, Harvard Medical School, Boston, MA, United States; ^2^ The Charles and Jane Pak Center for Mineral Metabolism and Clinical Research, University of Texas Southwestern Medical Center, Dallas, TX, United States; ^3^ Brigham and Women’s Hospital, Harvard Medical School, Boston, MA, United States; ^4^ Institute for Medical Engineering and Science, Massachusetts Institute of Technology, Cambridge, MA, United States; ^5^ Wyss Institute for Biologically-Inspired Engineering, Harvard University, Boston, MA, United States; ^6^ Endocrine Unit, Massachusetts General Hospital, Harvard Medical School, Boston, MA, United States; ^7^ Department of Mechanical Engineering, Boston University, Boston, MA, United States; ^8^ Department of Orthopaedic Surgery, Yerevan State Medical University, Yerevan, Armenia

**Keywords:** bone, fracture repair, nanoscale drug delivery, SIK2/SIK3 inhibitor, PTH, siRNA targeting, microparticle

## Abstract

**Introduction:**

Over 6.8 million fractures occur annually in the US, with 10% experiencing delayed- or non-union. Anabolic therapeutics like PTH analogs stimulate fracture repair, and small molecule salt inducible kinase (SIK) inhibitors mimic PTH action. This study tests whether the SIK inhibitor YKL-05-099 accelerates fracture callus osteogenesis.

**Methods:**

126 female mice underwent femoral shaft pinning and midshaft fracture, receiving daily injections of PBS, YKL-05-099, or PTH. Callus tissues were analyzed via RT-qPCR, histology, single-cell RNA-seq, and μCT imaging. Biomechanical testing evaluated tissue rigidity. A hydrogel-based delivery system for PTH and siRNAs targeting SIK2/SIK3 was developed and tested.

**Results:**

YKL-05-099 and PTH-treated mice showed higher mineralized callus volume fraction and improved structural rigidity. RNA-seq indicated YKL-05-099 increased osteoblast subsets and reduced chondrocyte precursors. Hydrogel-released siRNAs maintained target knockdown, accelerating callus mineralization.

**Discussion:**

YKL-05-099 enhances fracture repair, supporting selective SIK inhibitors’ development for clinical use. Hydrogel-based siRNA delivery offers targeted localized treatment at fracture sites.

## Introduction

Greater than 6.8 million fractures are reported annually in the United States, where approximately 10% will experience delayed- or non-union (Buza and Einhorn, 2016). In the United States, the median cost of treating fracture non-unions is estimated at $25,556 per open tibial fracture, with concomitant increases in healthcare resource utilization and increased prescription and longer duration of opioid use (Antonova et al., 2013). Bone fracture is associated with reduced functional independence, increased risk of future fractures, and increased morbidity and mortality (Bliuc et al., 2015; Marrinan et al., 2015). Osteoporosis is also a common worldwide disease with significant morbidity and mortality, leading to fractures at various skeletal sites, most often the spine, hip, or wrist (Barrett-Connor, 1995). Fifty percent of women and 20% of men aged over 50 will endure an osteoporotic fracture in their remaining lifetime (Nguyen et al., 2007). In 2005, there were more than 2 million osteoporosis-related fractures, costing nearly $17 billion. By 2025, annual fractures and associated costs are predicted to grow by 50%, to exceed 3 million and $25 billion, respectively (Burge et al., 2007).

Fracture healing involves the intricate coordination of cellular processes that partially recapitulate the normal process of endochondral bone formation (Einhorn and Gerstenfeld, 2015). Bone tissue is capable of repair without forming a fibrous scar. Four overlapping stages conventionally characterize fracture repair: the initial inflammation phase, soft callus (fibrocartilage) formation, hard callus formation (*i.e.*, mineralization), and callus remodeling (Roberts and Ke, 2018). Fracture repair time varies with specific fracture type and severity. However, most long bones heal in 6–8 weeks, and it is normal for vertebral fractures to heal in 8–10 weeks. This normal repair process sometimes fails, and non-unions or delayed unions occur (Marsell and Einhorn, 2010). FDA guidelines define non-union as a failure of bone repair after 9 months with no evidence of repair after three consecutive months (Khan et al., 2005). It is estimated that 100,000 non-unions occur annually in the United States. It is estimated that 5%–10% of all fractures will eventually form a non-union (Buza and Einhorn, 2016). This risk is even higher in individual bones, such as the tibia, with non-union rates of up to 18.5% (Fong et al., 2013). In addition to the direct costs that impaired fracture repair forces on patients and the healthcare system, the additional indirect financial cost of lost productive work time is significant and must be considered. Therefore, impaired fracture repair is a major problem associated with significant healthcare and societal costs, pain, and morbidity (Hak et al., 2014).

To reverse a non-union to a normal union, it is crucial to stimulate the intrinsic mechanisms of tissue repair (Roberts and Ke, 2018). Proper bone repair has many facets; mechanical stability and revascularization must be coordinated with osteogenesis, osteoinduction, and osteoconduction (Sohn and Oh, 2019). An autologous bone graft is the current “gold standard” to achieve this goal. The iliac crest is the most common harvesting site, with a complication rate of approximately 20% (Dimitriou et al., 2011). It faces several limitations and complications, including donor site pain, increased blood loss, morbidity, infection, and limited graft tissue availability, especially in pediatric patients (Goulet et al., 1997; Khan et al., 2005).

Since the advent of osteoanabolic drugs for the treatment of osteoporosis, systemic strategies for fracture repair have generated strong interest (Hegde et al., 2016). Teriparatide, abaloparatide, and romosozumab are the only FDA-approved bone anabolic agents to treat osteoporosis (Haas and LeBoff, 2018). Parathyroid hormone (PTH) is a central regulator of calcium metabolism (Potts, 2005). Intermittent pharmacologic hyperparathyroidism, achieved via once-daily subcutaneous injections, preferentially stimulates bone formation, boosts bone mass, and reduces fracture risk (Neer et al., 2001; Silva and Bilezikian, 2015; Miller et al., 2016). The effectiveness of teriparatide in promoting fracture healing has been demonstrated in several clinical case reports/series studies (Aspenberg et al., 2010; Mancilla et al., 2015). In a nonoperative treatment for tibial and femoral non-union, satisfactory healing was obtained with systemic therapy with teriparatide 20 μg/day for 8 months (Xiaofeng et al., 2017). Abaloparatide (a synthetic PTH/parathyroid hormone-related protein (PTHrP) hybrid peptide) treatment improved fracture repair in a rat closed fracture model (Lanske et al., 2019). PTHrP accelerates fracture healing in mice by enhancing callus formation and promoting cell differentiation (Wang et al., 2017). Although PTH analogs have not been approved for fracture repair augmentation, they are often prescribed “off-label” for this indication, limited by high cost, risk of hypercalcemia, and lack of proven efficacy (Stroup et al., 2008). Romosozumab is an anti-sclerostin antibody that enhances bone formation and decreases bone resorption (Padhi et al., 2011), with some evidence of fracture repair efficacy in preclinical models (Ominsky et al., 2011). None of these therapeutics are designed to offer local bone repair effects and may also produce undesired bone anabolic effects elsewhere in the skeleton. Thus, developing safe, tissue-selective, orally available therapies to promote fracture repair represents a significant opportunity for an unmet medical need.

The PTH intracellular signaling pathway in bone target cells involves protein kinase A-mediated inhibition of the cellular activity of salt-inducible kinases (SIKs) (Wein et al., 2016; Ricarte et al., 2018; Nishimori et al., 2019; Sato et al., 2021; Tang et al., 2021; Sato et al., 2022). Thus, direct small-molecule SIK inhibitors, such as YKL-05-099, mimic PTH actions *in vitro* and *in vivo*. Furthermore, SIK inhibitors also show anti-inflammatory actions (Babbe et al., 2024; Temal-Laib et al., 2024), thus differentiating this therapeutic approach from PTH treatment. We hypothesize that similar to intermittent PTH treatment, systemically administered YKL-05-099 accelerates fracture callus mineralization in mice. While systemically administered SIK inhibitor treatment may enhance fracture healing, novel approaches are needed to target this therapy specifically to sites of trauma. As such, we sought to develop methods for local delivery of siRNA-based therapy to reduce SIK2/SIK3 levels locally in the fracture callus.

## Results

**FIGURE 1:**
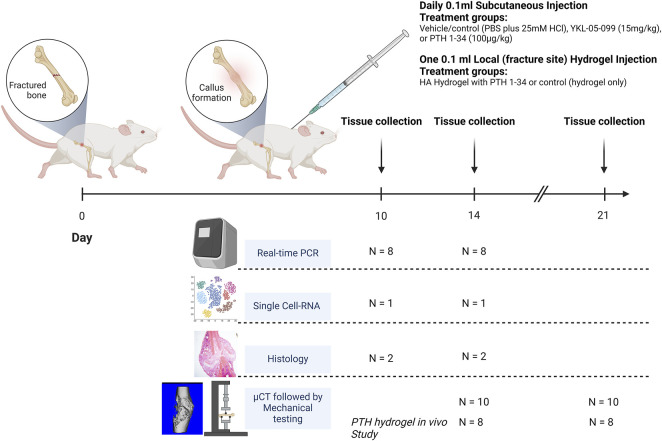
Animals were subjected to femoral fracture, with subsequent S.C treatment delivery (N = 42 per treatment group) or one-time Local delivery (N = 16 per treatment group). At 10 and 14 days post-operation, tissues were harvested from 8, 1, and 2 mice per treatment regimen for RT-qPCR, single-cell RNA, and histology, respectively. At 14- and 21-days post-operation, tissues were harvested from 10 animals for μCT and mechanical testing. In the PTH hydrogel *in vivo* study at 14- and 21 days post-operation, tissues were harvested from 8 animals for μCT testing.

**FIGURE 2:**
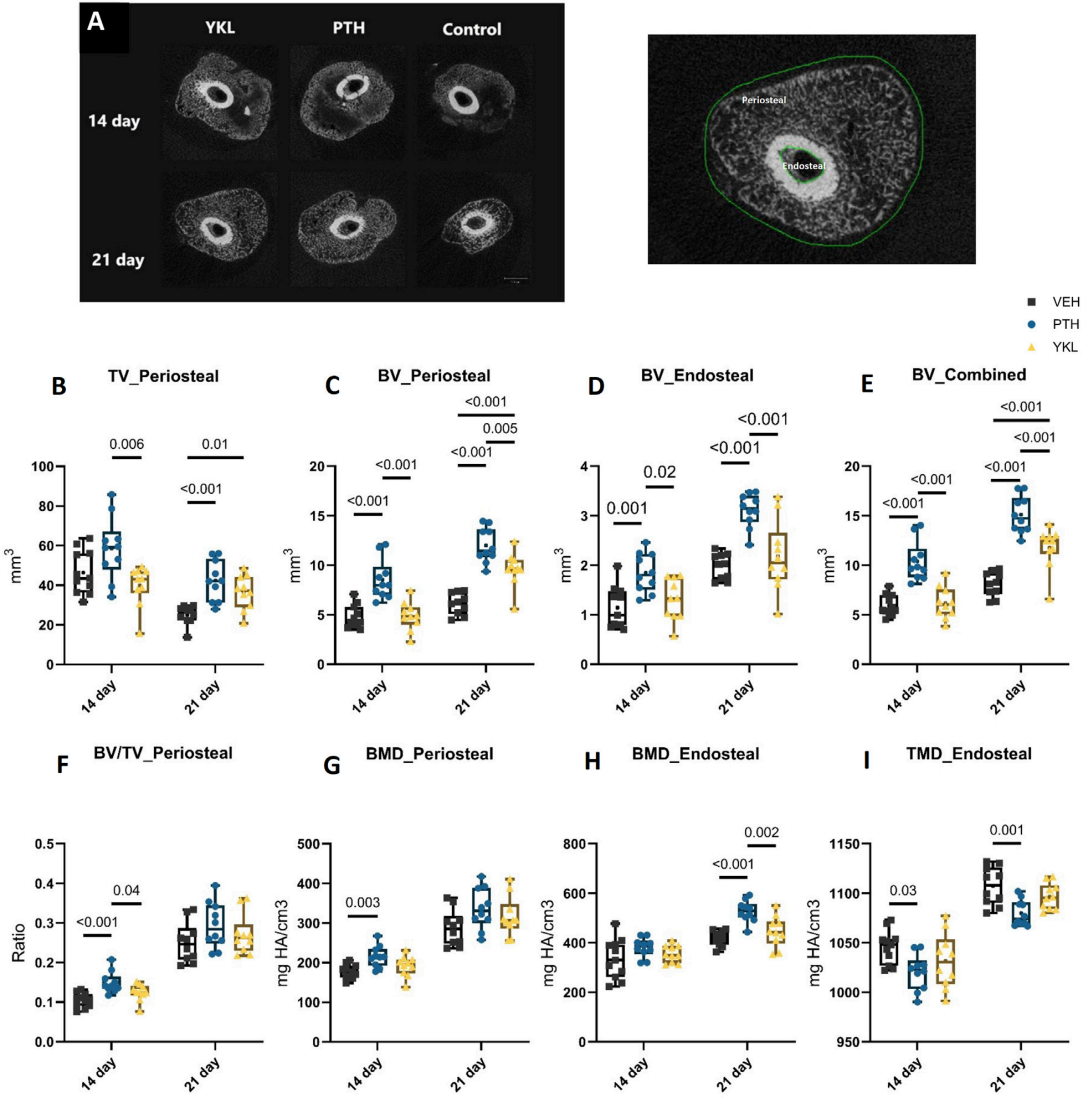
Panel **(A)** demonstrates axial images of the callus tissue and depicts periosteal and endosteal bone areas. **(B)** shows periosteal TV at 14 and 21 days. Panels **(C, D)** indicate periosteal and endosteal BV, respectively, while Panel **(E)** indicates combined BV. Panel **(F)** demonstrates the mineralized bone volume fraction (BV/TV). Panels **(G, H)** show periosteal and endosteal BMD, respectively. Panel **(I)** indicates endosteal TMD.

The study design is described above and summarized in Figure 1. Briefly, slow-healing C3H female mice (Jepsen et al., 2008) were subjected to midshaft femur fracture and then treated with either vehicle, PTH 1-34 (100 μg/kg/d), or YKL-05-099 (15 mg/kg/d, referred to subsequently as “YKL”) by once-daily subcutaneous injections. Utilizing μCT imaging, axial cut reconstructions in the middle of the callus tissues are shown in Figure 2A. μCT analysis revealed a significantly higher total volume of callus tissue (TV) in the PTH and YKL treatment groups compared to the control group at the 21-day timepoint in the periosteal region (Figure 2B). Furthermore, μCT analysis demonstrated increased mineralized bone volume (BV) in the PTH group at both 14 and 21-day time points in both periosteal and endosteal regions, compared to the control and YKL groups (Figures 2C,D). Notably, the YKL group exhibited significantly higher BV than the control group at the 21-day timepoint in the periosteal region and when combining periosteal and endosteal regions (Figures 2C,E). The fraction of mineralized callus volume (BV/TV) was significantly higher in the PTH group at the 14-day periosteal region timepoint than in the control and YKL groups. However, this significance was not maintained at the 21-day time in this region of interest (Figure 2F).

There was a significantly higher bone mineral density (BMD) for the PTH group at 14 days compared to the control group. Still, no significant difference in BMD was observed at the periosteal region at 21 days between the groups (Figure 2G). In the endosteal region, the PTH group showed significantly higher BMD at the 21-day timepoint. As for tissue mineral density (TMD), there were no significant differences at either timepoints between the groups (Figure 2H). However, in the endosteal region, the control group demonstrated a significantly higher TMD at both time points than the PTH and YKL groups (Figure 2I).

**FIGURE 3:**
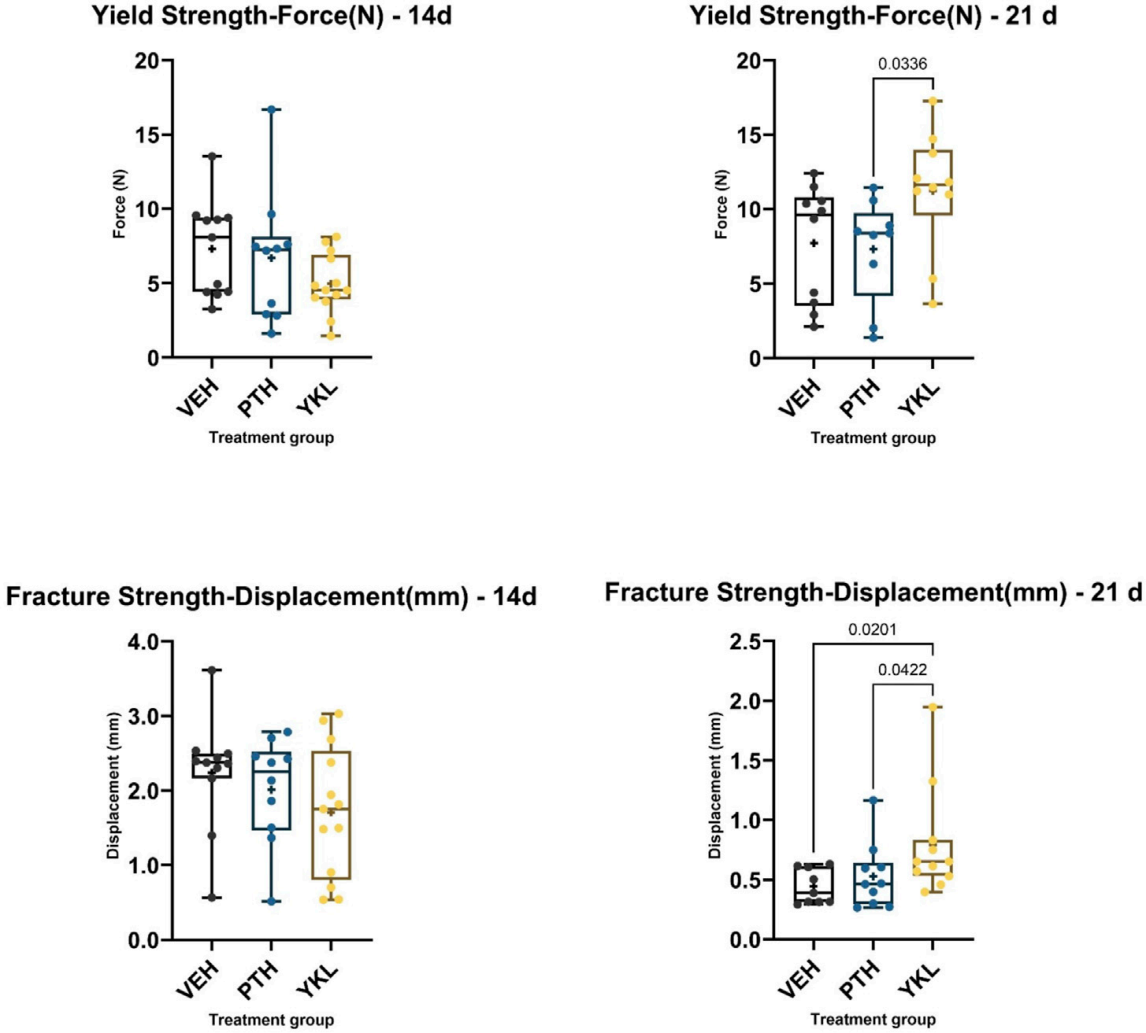
The panels demonstrate that YKL treatment showed superiority for the yield point load compared to the PTH group and for the fracture point displacement compared to the PTH and control groups.

Mechanical testing of harvested femurs was inconclusive at 14 days post-fracture due to considerable inter-animal variability. However, at 21 days, YKL treatment showed superiority for the yield load compared to the PTH group and for the fracture displacement compared to the PTH and control groups (Figure 3).

**FIGURE 4:**
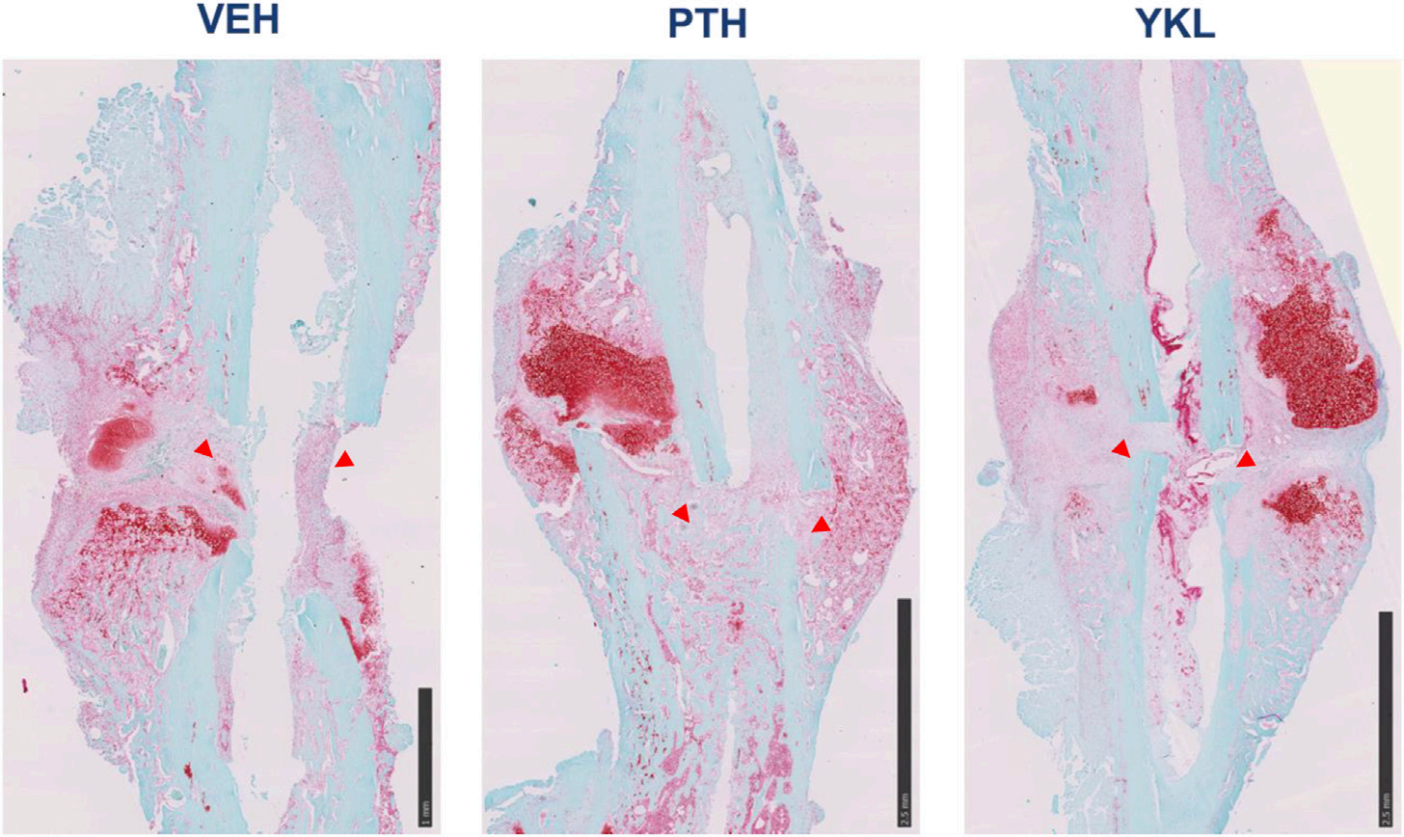
Safranin O-stained callus histology on day 14 post-operative, demonstrating that PTH and YKL treatment led to improved callus organization compared to the control treatment at this early point. Red arrowheads show increased osteogenic cells at the site of injury.

**FIGURE 5:**
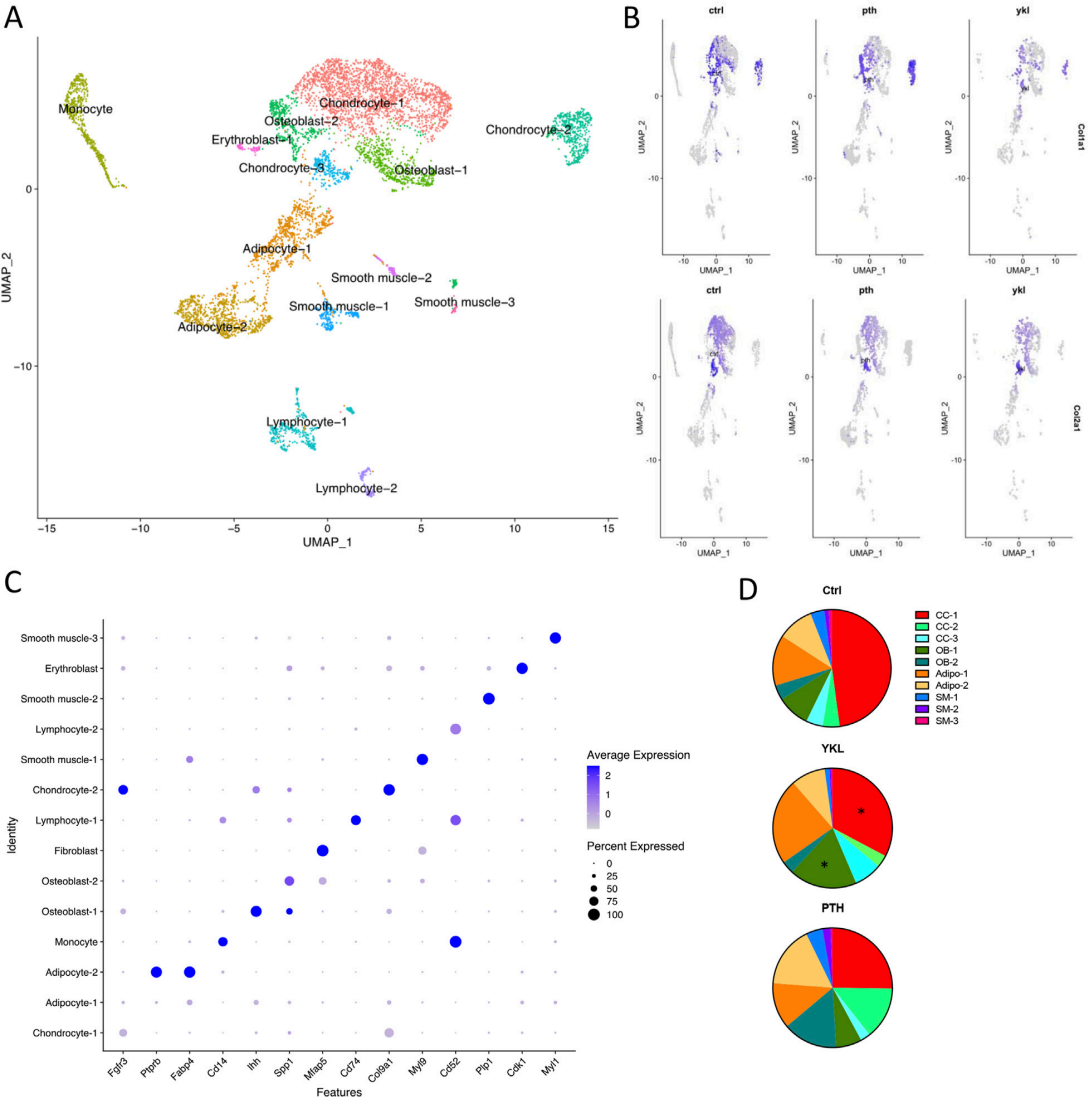
Callus tissue at day 14 was isolated for single-cell RNA sequencing to identify distinct groups of cells associated with fracture healing. The UMAP plot for integrated data is shown in panel **(A, B)** shows a data plot indicating each cluster’s relative expression of top marker genes. Feature plots depicting *Col1a1* (top) and *Col2a1* (bottom) expression in mice treated with vehicle (Ctrl), PTH, and YKL-05-099 are shown in panel **(C)**. In panel **(D)**, the relative proportion of non-immune cells was determined in different animals. YKL-05-099 treatment reduces the relative proportions of cells in chondrocyte cluster 1 (CC-1) and increases the relative numbers of osteoblast cluster 2 (OB-2) cells. Additionally, PTH treatment reduced the relative proportion of cells in CC-1 and did not increase the relative numbers in OB-2.

Hematoxylin and eosin-stained and Safranin O-stained sections of the callus 14 days post-fracture showed qualitative increases in osteoblast population and an accelerated transition from cartilaginous to the mineralized matrix in the groups treated with YKL-05-099 and PTH compared to vehicle-treated mice (Figure 4). Next, we isolated RNA from dissected callus tissue at different times to identify molecular correlates to the radiographic and histologic findings. At day 10, the expression of several osteoblast genes (*Spp1, Ibsp, and Bglap*) was qualitatively upregulated in response to YKL-05-099 treatment (Supplementary Figure S1). Due to variability in mRNA levels between experimental animals, the observed qualitative trends did not reach statistical significance. At day 14, PTH treatment led to statistically significant increases in *Spp1* expression, while the other genes profiled did not show statistically significant differences based on drug treatment groups (Supplementary Figure S2). Since the callus is a heterogeneous tissue with multiple types of cells present (Einhorn and Gerstenfeld, 2015; Wildemann et al., 2021), we reasoned that “bulk” RNA/RT-qPCR analysis may not be the ideal method to identify mechanisms of therapeutic action for PTH and SIK inhibitors in promoting callus mineralization. Therefore, we performed single-cell RNA sequencing of micro-dissected callus tissue. Similar to other studies reporting single-cell RNA sequencing of callus cells OBJ, this analysis revealed the presence of multiple molecularly defined subsets of mesenchymal lineage cells, including three chondrocyte clusters, two osteoblast clusters, and two adipocyte clusters (Figure 5A). The top marker from each cluster was identified (Figure 5B), such as *Fgfr3* (chondrocyte-1), *Col9a1* (chondrocyte-2), *Ihh* (osteoblast-1) and *Spp1* (osteoblast-2). Feature plots of bone markers further showed that *Col1a1* expression is reduced while *Col2a1* is increased in chondrocyte subpopulations (Figure 5C). Compared to the control, PTH and YKL treatments reduced the number of cells in chondrocyte cluster 1 (CC-1). YKL increased the relative number of osteoblast cluster 2 (OB-2) cells while PTH did not (Figure 5D). Thus, single-cell RNA sequencing further supports the observation that systemic YKL-05-099 treatment enhances callus mineralization.

**FIGURE 6:**
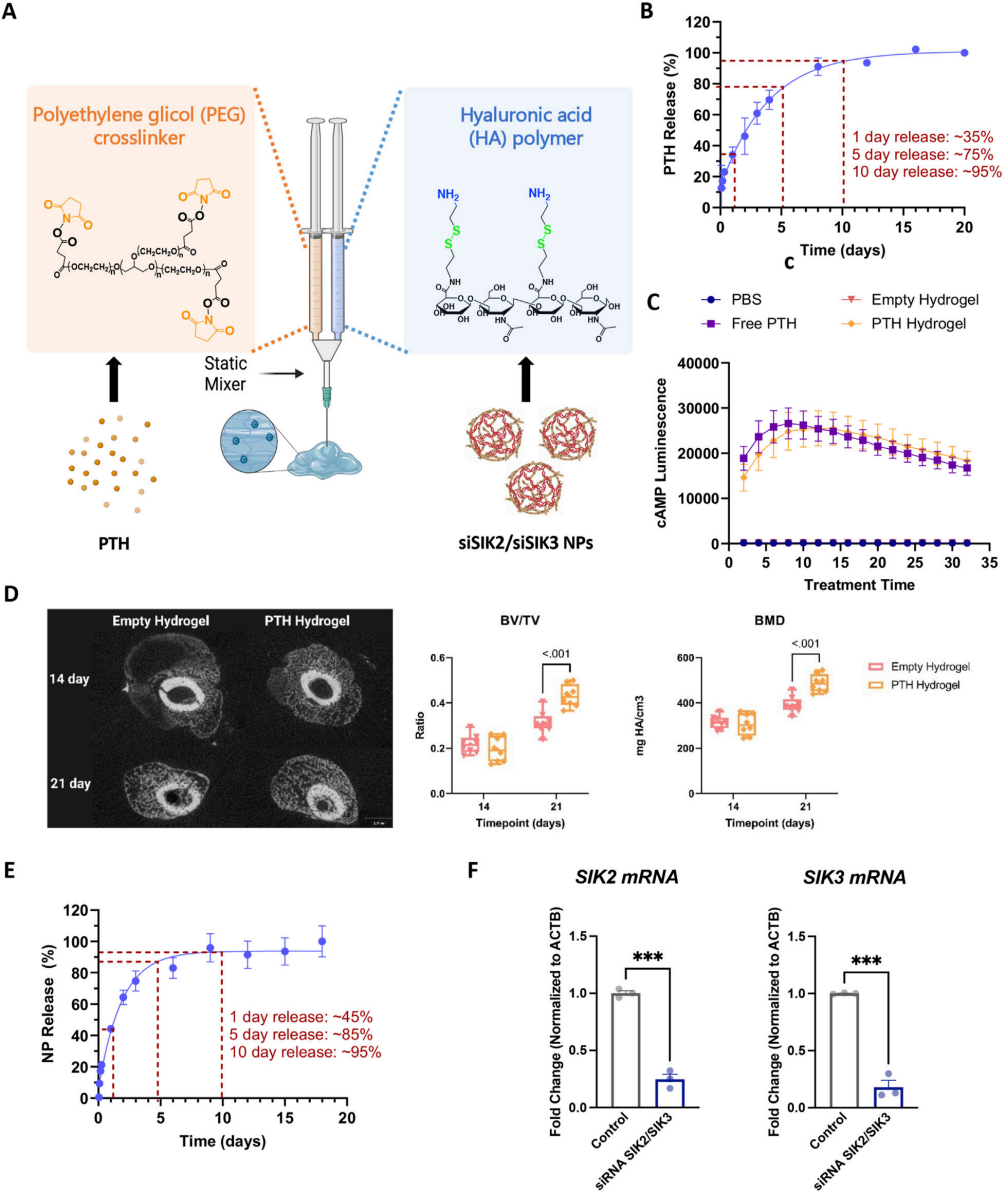
Hyaluronic Acid-based injectable hydrogel for the local delivery of PTH 1-34 and siSIK2/siSIK3 NPs. **(A)** Hydrogel formation. Amine-modified HA solution is crosslinked using the NHS-terminated 8-arm PEG crosslinker. PTH 1-34 is loaded to the NHS-terminated 8-arm PEG crosslinker, and siSIK2/SIK3 NPs are loaded to the amine-modified HA. **(B)** Cumulative release of PTH1-34 from HA hydrogel over 20 days *in vitro*. **(C)** *In vitro* functional study of PTH 1-34 released from the hydrogel. **(D)** *In vivo* local delivery of PTH 1-34 improved callus mineralization. **(E)** Cumulative release of siSIK2/siSIK3 NPs from HA hydrogel over 20 days *in vitro*. **(F)** *In vitro* functional study of siSIK2/siSIK3 NPs released from the hydrogel. All data are represented as mean ± SD; n = 3. Panel A was created with BioRender.com.

Prompted by these promising findings with systemically administered SIK inhibitors, we sought to develop an approach to locally target SIKs at the fracture site. A hyaluronic acid (HA)-based injectable hydrogel was developed to enable efficient and local SIK-targeting agent delivery. The hydrogel employed here is spontaneously formed by the reaction between the HA primary amines and the 8-arm-PEG-NHS crosslinker containing a succinimidyl functional group (Figure 6A). Before developing this system for SIK targeting agents, we first asked whether parathyroid hormone itself could be loaded. *In vitro* release studies confirmed that parathyroid hormone 1–34 (PTH) is released from the HA in two distinct phases. 35% of PTH is released during the first 24 h following a sustained release of up to 10 days, where 95% of PTH is released (Figure 6B). We also confirmed that PTH 1–34 activity is not affected by loading into HA hydrogels using PTH receptor-expressing HEK293 cells bearing a cAMP-sensitive sensor as a sensitive bioassay. PTH released from HA hydrogels retains comparable bioactivity to ‘free’ PTH peptides without hydrogel loading (Figure 6C). Consistent with previous reports (Wojda et al., 2020; 2021; Li et al., 2023; Long et al., 2023), local delivery of PTH via hydrogel to the fracture site improved callus mineralization (Figure 6D).

**FIGURE 7:**
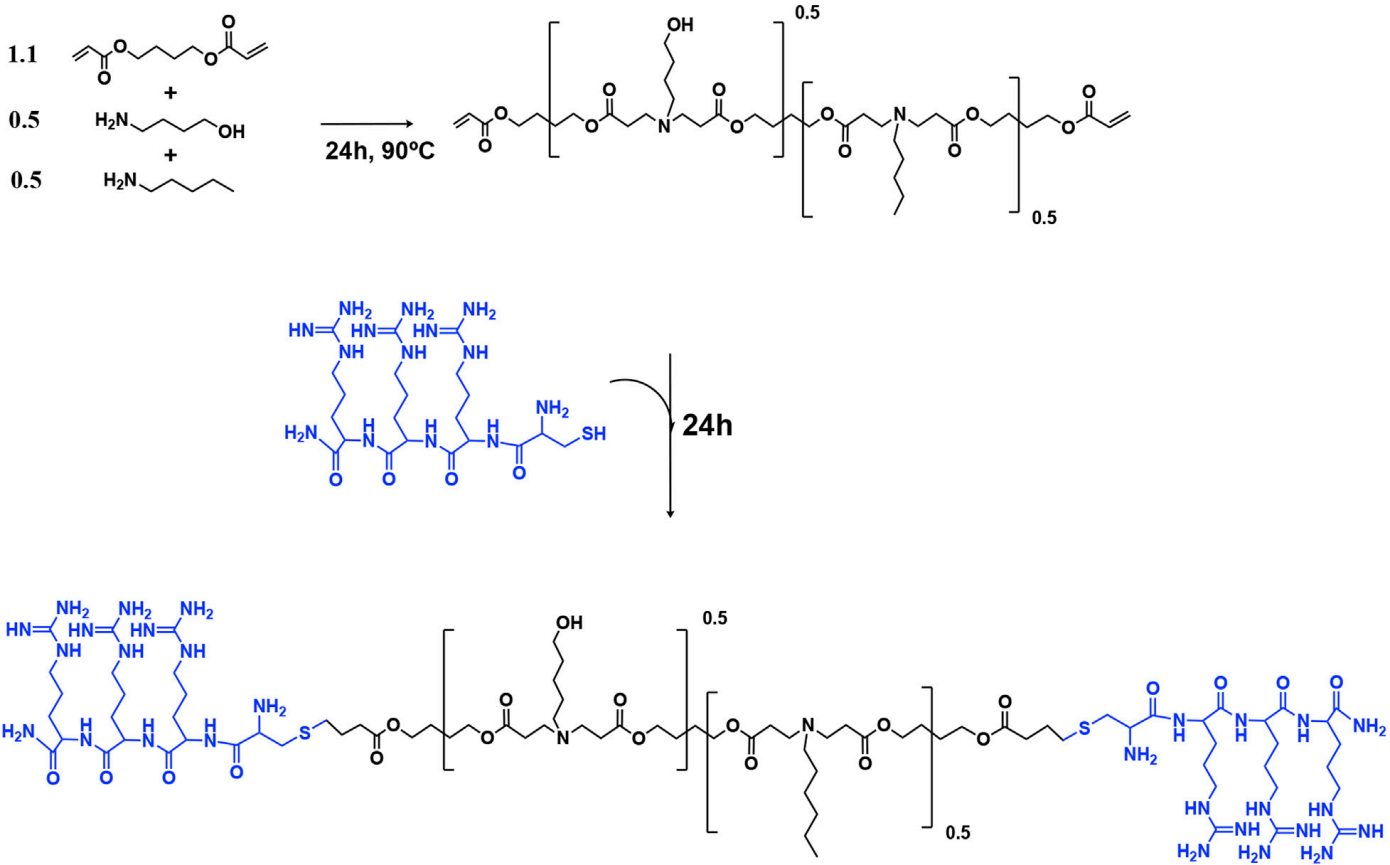
Synthesis of arginine modified poly (beta-amino ester)s (pBAEs) polymer. The acrylate-terminated polymer was synthesized by the additional reaction of primary amines with diacrylates. Then, the arginine-modified pBAEs were obtained by end-capping modification of the resulting acrylate-terminated polymer with Cys-Arg-Arg-Arg peptide. Their chemical structures were characterized by 1H-NMR.

**FIGURE 8:**
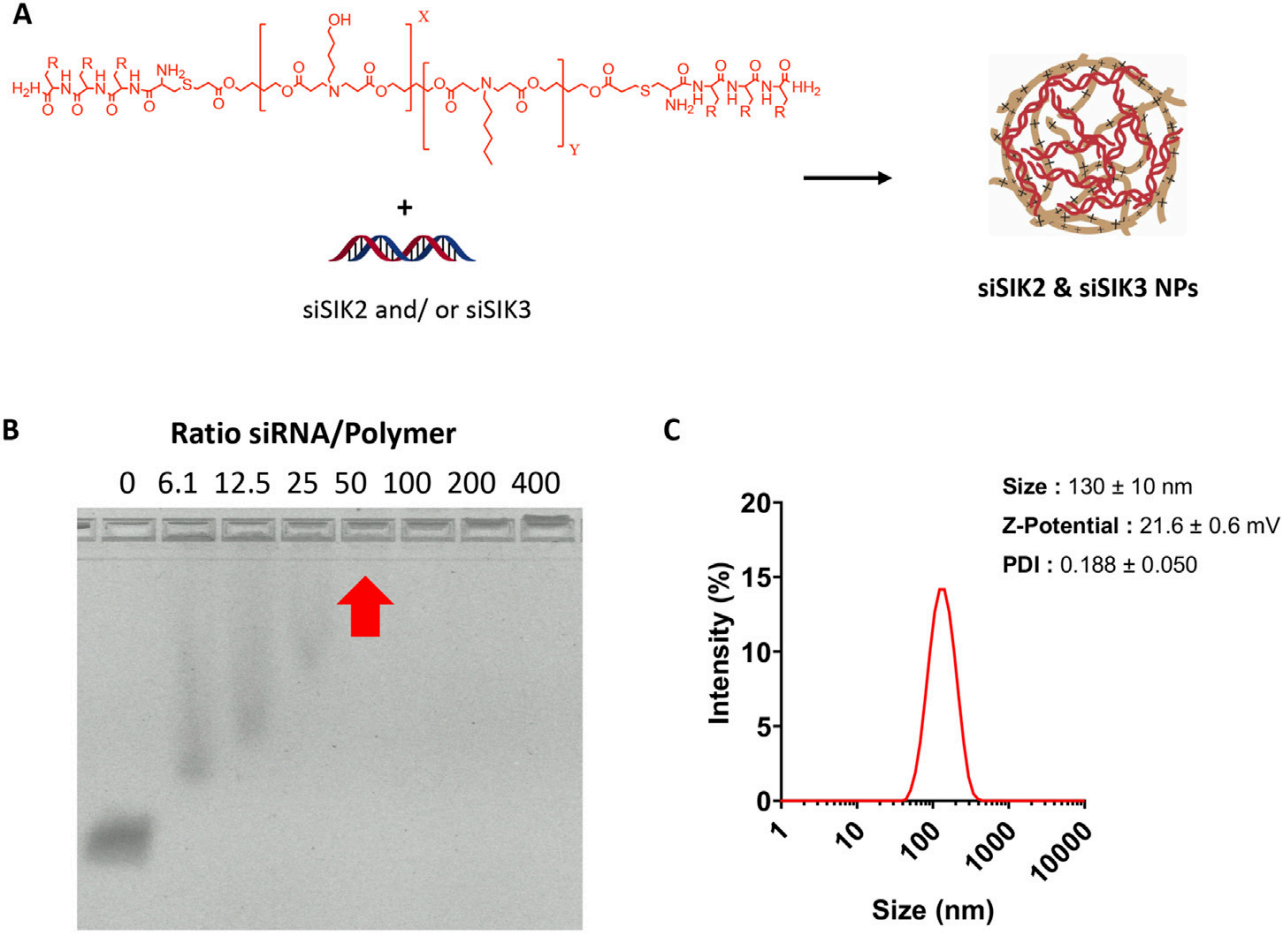
Optimization and characterization of siSIK2 and SIK3 NPs. **(A)** Scheme of arginine-modified pBAE complexation with siRNA. **(B)**, Agarose retardation assay of arginine-modified pBAE complexed with siRNA. NPs were formed using siSIK2 and arginine-modified pBAE at indicated w/w ratios and loaded onto an agarose gel to assess siSIK2 mobility by electrophoresis. **(C)** Size, Z-potential, and polydispersity (PDI) of complexed siRNA using arginine-modified pBAEs were determined by DLS.

Having established the therapeutic efficacy of this hydrogel system in mouse fracture healing, we next sought to target SIK2 and SIK3 at the fracture site. We chose these two SIK isoforms based on our previous mouse genetic data, demonstrating robust increases in bone formation in uninjured bones following the deletion of these two SIK genes (Nishimori et al., 2019; Tang et al., 2021). We employed a siRNA-based genetic approach to avoid off-target effects ascribed to pharmacologic SIK inhibitors. siRNAs targeting murine SIK2 and SIK3 genes were encapsulated using poly (beta-amino acid) (pBAE) polymers (Figure 7). A weight:weight ratio of 50:1polymer:siRNA was needed to encapsulate all the siRNA, resulting in a nanoparticle size of 130 ± 10 nm with a surface charge of 20.6 ± 0.6 mV (Figure 8). siSIK2/SIK3 NPs led to robust target knockdown in murine MC3T3-E1 osteoblastic cells (Supplementary Figure S3). In addition, *in vitro* release studies showed that siSIK2/siSIK3 NPs are released from the HA following release kinetics similar to that of PTH 1-34 (Figure 6E), and similar *in vitro* target knockdown results were observed when siRNA/nanoparticle complexes were recovered following release from HA hydrogels (Figure 6F). These results suggest that local genetic targeting of SIK2 and SIK3 at the fracture site may be possible via this nanoparticle/hydrogel delivery system.

## Discussion

We show that YKL-05-099, a small molecule SIK inhibitor, can effectively stimulate fracture repair in a slow-healing mouse model. This finding is noteworthy because it introduces a potential alternative to current treatment methods, such as autologous bone grafts, which have limitations and complications. Compared to PTH injections, small molecule SIK inhibitors possess several distinct advantages. First, small molecules are more likely to show robust and consistent oral availability, raising the possibility for more convenient oral therapy. Second, it is easy to envision how bone-targeting strategies such as bisphosphonate or oligo-aspartate conjugation could readily target a small molecule at a fracture site. Indeed, bone-targeting of dasatinib, a small molecule kinase inhibitor that potently blocks SIKs (Ozanne et al., 2015), stimulates fracture healing in mice (Wang et al., 2018). Third, unlike PTH, salt-inducible kinases also function in other cAMP-linked signaling systems and exert anti-inflammatory actions (Clark et al., 2012; Sundberg et al., 2014; Babbe et al., 2024). Therefore, it is possible that optimized SIK inhibitor-based strategies, by simultaneously stimulating bone formation and suppressing inflammation, may exert actions distinct from PTH-based fracture healing modalities.

Several strategies and various pharmacological interventions have been used to enhance bone fracture repair (Einhorn and Gerstenfeld, 2015; Ghiasi et al., 2017; Roberts and Ke, 2018; Calcei and Rodeo, 2019). However, optimal stimulation of fracture repairs remains a holy grail in orthopedic research**.** Fracture repair can be enhanced by biological or biophysical means (Einhorn and Gerstenfeld, 2015). Biophysical stimulation to promote fracture repair still needs to be studied at present (Wright et al., 2005; Busse et al., 2009). Medical therapies can be grouped into local and systemic treatments (Einhorn and Gerstenfeld, 2015). Currently, BMP2-infused bone graft (rhBMP2 + Absorbable collagen sponge as a scaffold, Medtronic) is the only FDA-approved treatment for fracture repair (Garrison et al., 2007; Papakostidis et al., 2008; Hustedt and Blizzard, 2014). Since its approval, many researchers have re-examined its clinical use and reported negative results. BMP2 is criticized due to side effects (Devine et al., 2012; Gross, 2012; Roberts and Rosenbaum, 2012; Bray et al., 2017), significant postoperative morbidities and mortalities, and high price. BMP7 (Ossigraft, Stryker, Canton, MA USA) failed to obtain FDA premarket approval due to limited efficacy (White et al., 2007; Vaccaro et al., 2008). A rapid burst of drug release may contribute to unwanted side effects related to local administration. Given that PTH signaling leads to SIK inhibition, we tested whether small molecule SIK inhibitors such as YKL-05-099 (Sundberg et al., 2016; Mujahid et al., 2017) might mimic PTH action in bone. SIK inhibitor treatment mimics the effects of PTH *in vitro* and *in vivo* to a remarkable degree based on highly concordant phenotypic and gene expression changes (Wein et al., 2016; Nishimori et al., 2019; Yoon et al., 2023).

One of the key findings of this study is the improved mechanical response observed with YKL-05-099 treatment during the later phases of healing. Bones treated with YKL-05-099 demonstrated better mechanical properties, requiring more force to deform, and exhibited greater resistance to displacement. These enhanced mechanical properties are crucial for healed fractures’ functional recovery and long-term stability. Histological analysis showed qualitative improvements in callus organization in the YKL-05-099 and PTH treatment groups at an early time point. This suggests that these treatments promote a more efficient transition from cartilaginous to mineralized matrix, essential for proper bone healing. scRNA-seq analysis revealed that PTH and YKL-05-099 treatments reduced chondrocytic gene expression (Col2a1) while increasing osteoblastic gene expression (Col1a1). This shift in gene expression profile aligns with the desired outcome of promoting bone formation over cartilage formation during fracture repair. Single-cell RNA sequencing provided valuable insights into the cellular composition of the fracture callus tissue. YKL-05-099 treatment was associated with a reduction in chondrocyte-like precursors and an increase in subsets of osteoblasts. This information helps elucidate the specific cellular mechanisms underlying the improved fracture repair observed with YKL-05-099. While we did not assess osteoblast or osteoclast activity histologically in this study, suppressing osteoclast function at a high YKL dose has been shown previously (Tang et al., 2021).

In the dynamic process of bone healing, the YKL treatment group demonstrated promising results, particularly in endochondral ossification, integral to the periosteal region’s recovery. The μCT data reveals that YKL-05-099 fosters a conducive environment for bone regeneration, as evidenced by the tissue volume (TV) and mineralized bone volume (BV) parameters. Despite the superior performance of PTH in certain aspects, YKL shows a consistent trend toward improving bone healing at critical time points. Notably, the periosteal region—crucial for endochondral bone formation—has responded favorably to SIK inhibitor treatment, indicating its potential role in enhancing the periosteal callus formation. These findings suggest that SIK inhibitors like YKL-05-099 could be a beneficial therapeutic approach for fractures.

While we did not observe any off-target behavioral or tissue effects for YKL-05-099, this study only focused on callus tissue; further research is needed. Previous studies using higher doses of YKL-05-099 noted small increases in serum BUN and glucose with long-term treatment (Tang et al., 2021). Future studies must test the effects of hydrogel-delivery locally-delivered SIK inhibitors and/or SIK2/SIK3 siRNA in fracture healing and distant organ tissue analysis. Additionally, demonstrating *in vivo* target engagement in bone tissues is a major challenge for pharmacologic studies with kinase inhibitors since phosphorylation of intracellular proteins can be quite labile, is difficult to detect by immunostaining, and only sometimes persists during protein isolation protocols for immunoblotting. We previously demonstrated target engagement by YKL-05-099 based on gene expression changes in bone (Wein et al., 2016), and others demonstrated that this drug reduces SIK substrate phosphorylation levels in the spleen (Sundberg et al., 2016).

The multifaceted influence of SIK inhibitors on various cell types within the fracture callus, particularly promoting osteoblast differentiation, is a promising avenue for advancing fracture healing therapies. Further development of potent/selective small-molecule SIK inhibitors and genetic strategies for local SIK2/SIK3 inhibition at sites of skeletal injury is needed to translate these findings into clinical applications. The findings presented in this study underscore the importance of exploring innovative approaches to fracture repair beyond traditional methods. Targeting specific cellular pathways and optimizing bone healing will improve clinical outcomes, reduce complications, and alleviate costs associated with impaired fracture repair.

## Methods

Animal procedures were approved by the Beth Israel Deaconess Medical Center Institutional Animal Care and Use Committee (IACUC).

### Surgical procedure

One hundred fifty-eight (of which thirty-two for the PTH hydrogel *in vivo* study) nine-week-old female C3H/HeJ mice (Charles River, Inc., Wilmington, MA), a strain selected due to slow fracture healing characteristics (Jepsen et al., 2008), were subjected to a unilateral closed femoral fracture using a drop weight method and intramedullary fixation described previously (Marturano et al., 2008; Wixted et al., 2009). Briefly, the central cannula from a 22-gauge spinal needle was inserted into the femoral medulla retrograde through a medial parapatellar arthrotomy. The femur was held in a fixed position while a drop weight from a standard height was used to deliver a fixed traumatic injury to the mid-portion of the femur, generating a fracture via three-point bending.

### Treatment groups

The animals were treated with daily 0.1 mL subcutaneous (S.C.) injections of vehicle/control (PBS plus 25 mM HCl), YKL-05-099 (15 mg/kg, described in (Sundberg et al., 2016)), or PTH 1-34 (100 μg/kg, synthesized by the MGH peptide core as in (Tang et al., 2021)), starting on the day of surgery. PTH hydrogel *in vivo* study animals received a one-time injection of 0.1 mL HA hydrogel with PTH 1-34 (80 μg per injection) or control HA hydrogel injected locally over the femoral fracture site. Animals were euthanized at 10, 14, and 21 days postoperatively, and the fractured femurs were harvested (Figure 1). The sample size was determined by power calculation based on preliminary data with power = 0.8 and a = 5%. The harvested femurs were either fixed in 10% neutral buffered formalin for histological studies or 70% ethanol for micro-computed tomography (μCT), and mechanical testing. In the remaining specimens, using a sterile technique, fresh callus tissue was dissected, placed in TRIzol reagent, and frozen in liquid nitrogen. These samples were stored at −80°C for subsequent single-cell RNA sequencing or real-time qPCR analysis.

### μCT imaging and CT-based rigidity analysis

Sequential transaxial images through the entire femur were obtained by a desktop μCT system (μCT 40; Scanco Medical AG, Brüttisellen, Switzerland) using 70 kVp peak tube voltage, 114 μA tube current, 250 ms integration time, and 10-μm voxel size. The fracture site and callus region were identified and contoured, and periosteal and endosteal callus formation was distinguished and analyzed separately. A global threshold of 462.0 mg HA/cm^3^ was used to define mineralized callus in the periosteal space, while a threshold value of 659.3 mg HA/cm^3^ was used for the endosteal space. A Gaussian filter (sigma = 0.8, support = 1.0) was applied for noise reduction. Total callus volume (TV), mineralized callus volume (BV), mineralized callus volume fraction (BV/TV), tissue mineral density (TMD), and bone mineral density (BMD) were evaluated using the built-in software.

### Mechanical testing

Following μCT imaging, femurs were subjected to three-point bending to failure using a Bose Electroforce 3200 (TA Instruments, Wintest v4.1, Wakefield, MA, United States) testing apparatus. Before testing, the femurs were soaked in a phosphate buffer solution for 24 h. Care was taken to position the bones horizontally with the anterolateral surface facing downward, centered on the support span 8 mms apart. Before the actual testing, a small stabilizing preload of 0.5 N was applied on the posterior surface of the femur at a rate of 0.2 mm/s. Then, the pressing force was directed perpendicular to the midshaft of the bone with a constant speed of 0.03 mm/sec until failure and force-displacement data were collected every 0.03 s. Tissue biomechanical properties such as stiffness, load, and displacement for Yield, Ultimate, and fracture points were extrapolated from the load-deformation curves.

### Histology

Specimens were fixed in 10% neutral buffered formalin for 48 h. The fixed specimens were then decalcified in EDTA for 2 weeks, processed with ethanol and xylene, and embedded in paraffin on an automatic processor. Paraffin blocks of each specimen were then sectioned along the long axis of the bone. Sections of 5 μm thickness were baked on glass slides at 60°C and then stained with hematoxylin and eosin or Safranin-O.

### Real-time qPCR

After removing all overlying soft tissue, dissected calluses were placed in TRIzol reagent and stored at −80°C. RNA was extracted by tissue homogenizer with TRIzol (Life Technologies, Carlsbad, CA, United States) following the manufacturer’s instruction, and further purification was performed with PureLink RNA mini-column. cDNA was prepared with 1 µg RNA and synthesized using the Primescript RT kit (Takara Bio, San Jose, CA, United States). qPCR assays were performed on the StepOnePlus™ Real-time PCR System (Applied Biosystems, Waltham, MA United States) using SYBR Green FastMix ROX (Quanta bio, Beverly, MA, United States). *β*-actin was used as the internal control for normalization. The 2^−ΔΔCT^ method was used to detect expression fold change for each target gene.

### Single cell RNA sequencing

Seven days post-operation, callus tissue was meticulously harvested. A dissection approach was employed, deliberately avoiding the bone marrow to minimize hematopoietic cell carryover. The callus material was subjected to collagenase digestion (0.2% collagenase type I, 35°C/15 min/500 rpm) to obtain a single-cell suspension. Single cells were encapsulated into emulsion droplets using a Chromium Controller (10× Genomics, Pleasanton, CA, United States). scRNA-seq libraries were constructed using Chromium Single-Cell 3′ v3 Reagent Kit according to the manufacturer’s protocol. Raw reads obtained from scRNA-seq experiments were demultiplexed, aligned to the mouse genome, version mm10 (with tdTomato gene inserted), and collapsed into unique molecular identifiers (UMIs) with the Cellranger toolkit (10 × Genomics, version 3.1.0). The Seurat object was made with the count matrix for quality control, normalization, clustering, and identification of differentially expressed genes.

### Modified hyaluronic acid (HA) synthesis

The amine-modified HA was synthesized following a previously described procedure (Dosta et al., 2023). 60 kDa sodium hyaluronate was dissolved at 1% w/v in MES buffer and activated using N-(3-(dimethylamino)propyl)carbodiimide (EDC) and N-hydroxysuccinimide (NHS) at a 1:4:2 molar ratio. The reaction was performed at room temperature for 30 min. The activated hyaluronic acid (HA) was reacted with cysteamine dihydrochloride at a 1:10 molar ratio and allowed to react at room temperature for 12 h. The resulting product underwent purification via dialysis, followed by freeze-drying, and was finally stored at −20°C. The molecular structure of amine-modified HA was analyzed using 1H-NMR with D2O as a solvent (400 MHz Varian NMR spectrometer).

### Hyaluronic acid injectable delivery system

Amine-modified hyaluronic acid was dissolved in phosphate buffer (pH = 7.4) containing the PTH to achieve a 10% (w/v) HA solution. Simultaneously, 8-arm-PEG-NHS crosslinker was dissolved in phosphate buffer (pH = 7.4) to achieve a 10% (w/v) solution. For *in vitro* experiments, the two polymer solutions were vigorously mixed together inside cylindrical plastic molds. The resulting hydrogel disks were allowed to react for 5 min to ensure complete gelation. For *in vivo* experiments, the two polymer solutions were loaded into a double-channel syringe coupled to a mixer, facilitating hydrogel formation upon injection.

### Synthesis of arginine-modified poly-beta-amino-esters (pBAE)

Synthesis of pBAEs was performed via a two-step procedure, as previously described (Dosta et al., 2015; Dosta et al., 2018). An acrylate-terminated polymer was obtained by the addition reaction of 5-amino-1-pentanol (0.5 mmol) and hexylamine (0.5 mmol) to 1,4-butanediol diacrylate (1.1 mmol). The reaction was carried out at 90°C for 24 h. Then, the acrylate-terminated polymer was end-capped with thiol-terminated arginine peptide (Cys–Arg–Arg–Arg) at a 1:2.1 molar ratio in dimethyl sulfoxide (DMSO). The resulting polymer was purified by precipitation in a mixture of diethyl ether and acetone (7:3). pBAE polymer structure was confirmed by 1H-NMR (400 MHz Varian (NMR Instruments, Clarendon Hills, IL, United States)).

### siSIK2 and siSIK3 NP formation and characterization

siSIK2 and siSIK3 NP were performed by mixing equal volumes of pBAEs polymer and siRNA in 12.5 mm acetate buffer (AcONa) at their appropriate concentration. The pBAE polymer was added to a solution of siRNA, incubated at room temperature for 15 min, and precipitated in PBS 1×. The resulting nanoparticles were characterized by an agarose retardation assay and dynamic light scattering (DLS). To assess siRNA retardation, different siRNA-to-polymer ratios (w/w), ranging from 6 to 400, were studied. siRNA NPs were prepared and loaded in 2% E-Gel Precast Agarose Gels (Thermo Fisher), run following the manufacturer’s instructions, and visualized in fluorescence mode. Biophysical characterization of nanoparticles was performed using a ZetaSizer Nano ZS equipped with a He–Ne laser (*λ* 1/4,633 nm) at a scattering angle of 137° (Malvern Instruments Ltd., Malvern, United Kingdom). Hydrodynamic diameter (nm), PDI, and surface charge of nanoparticles were measured.

### siRNA NP and PTH release study

HA hydrogel disks containing siRNA NPs or PTH were formed in cylindrical plastic molds (diameter: 5.00 mm; height: 2.50 mm). The hydrogel disks were placed in a 24-well plate with 1 mL of PBS and incubated at 37°C in the dark. For the selected time points, 100 mL aliquots of the release medium were taken for further analysis. PTH release was quantified by measuring the fluorescence intensity (λ_ex_ = 550 nm; λ_em_ = 590 nm) using a multimodal plate reader (TECAN).

### siSIK2 and siSIK3 knockdown study

Murine MC3T3-E1 osteoblastic cells were seeded in 12-well plates at 150,000 cells/mL, incubated overnight to roughly 80% confluence, and treated using siSIK2/SIK3 NPs. Transfections were carried out with 10% FBS in the transfection medium for 48 h. SIK2 and SIK3 mRNA levels were analyzed by qPCR. siRNA-SCR was used as a negative control.

### PTH *in vitro* study

HEK293 cells stably expressing the human PTH1R and the Glosensor cAMP reporter were maintained in DMEM (10% FBS, 1% penicillin/streptomycin). Cells were seeded into 96-well white plates (150,000 cells/mL). 24–48 h after confluency, the wells were rinsed with 100 μL carbon dioxide-independent culture medium (CIDB; Life Technologies) containing 0.1% bovine serum albumin (BSA), which was then replaced with 90 μL of luciferin (0.5 mM in CIDB) for 30 min before adding treatments: vehicle (PBS), free PTH 1-34 (10^−7^M), empty hydrogel, or hydrogel-released PTH. The assay was performed at room temperature. cAMP-dependent luminescence was measured at 2-min intervals using PerkinElmer Envision Plate reader. The luminescence values were plotted versus time using GraphPad Prism 9.2. Ligand response curves were generated as described (Guo et al., 2017).

### Statistical analysis

The Shapiro-Wilk test was used to assess the normality of the data for each variable. Based on the normality tests, a one-way analysis of variance (ANOVA) or Kruskal–Wallis H test was conducted to compare the effect of treatment on the outcome of interest. Post hoc pairwise comparisons were made using the Tukey-Kramer and Dunn’s tests for ANOVA and Kruskal–Wallis H tests, respectively. Two-tailed *p*-values less than 0.05 were considered significant. All statistical analysis was performed using GraphPad Prism (version 9.3.0 for Windows; GraphPad Software, San Diego, CA, United States) (Supplementary Table S1).

## Data Availability

The datasets presented in this study can be found in online repositories. The names of the repository/repositories and accession number(s) can be found in the article/Supplementary Material.

## Supplementary material

SUPPLEMENTARY FIGURE S1 Gene expression changes as expressed from the callus tissue of the fracture site at ten days.

SUPPLEMENTARY FIGURE S2 Gene expression changes as expressed from the callus tissue of the fracture site at 14 days.

SUPPLEMENTARY FIGURE S3 SIK2 and SIK3 silencing using arginine-modified polymer. Levels of SIK2 (A) and SIK3 (B) were determined by qPCR. Data are represented as mean ± SEM (n = 3). The data was analyzed by one-way ANOVA with Bonferroni’s multiple-comparisons tests.

SUPPLEMENTARY Table 1A P values for volumetric uCT analysis of 14- and 21-day timepoints.

SUPPLEMENTARY TABLE 1B P values for uCT-based density analysis of 14- and 21-day time points.
