# Supplementary figures and images for: Stimulation of fracture mineralization by salt-inducible kinase inhibitors
Source: Front Bioeng Biotechnol. 2024 Sep 16;12:1450611. doi: 10.3389/fbioe.2024.1450611 (PMC11445660; doi:10.3389/fbioe.2024.1450611)

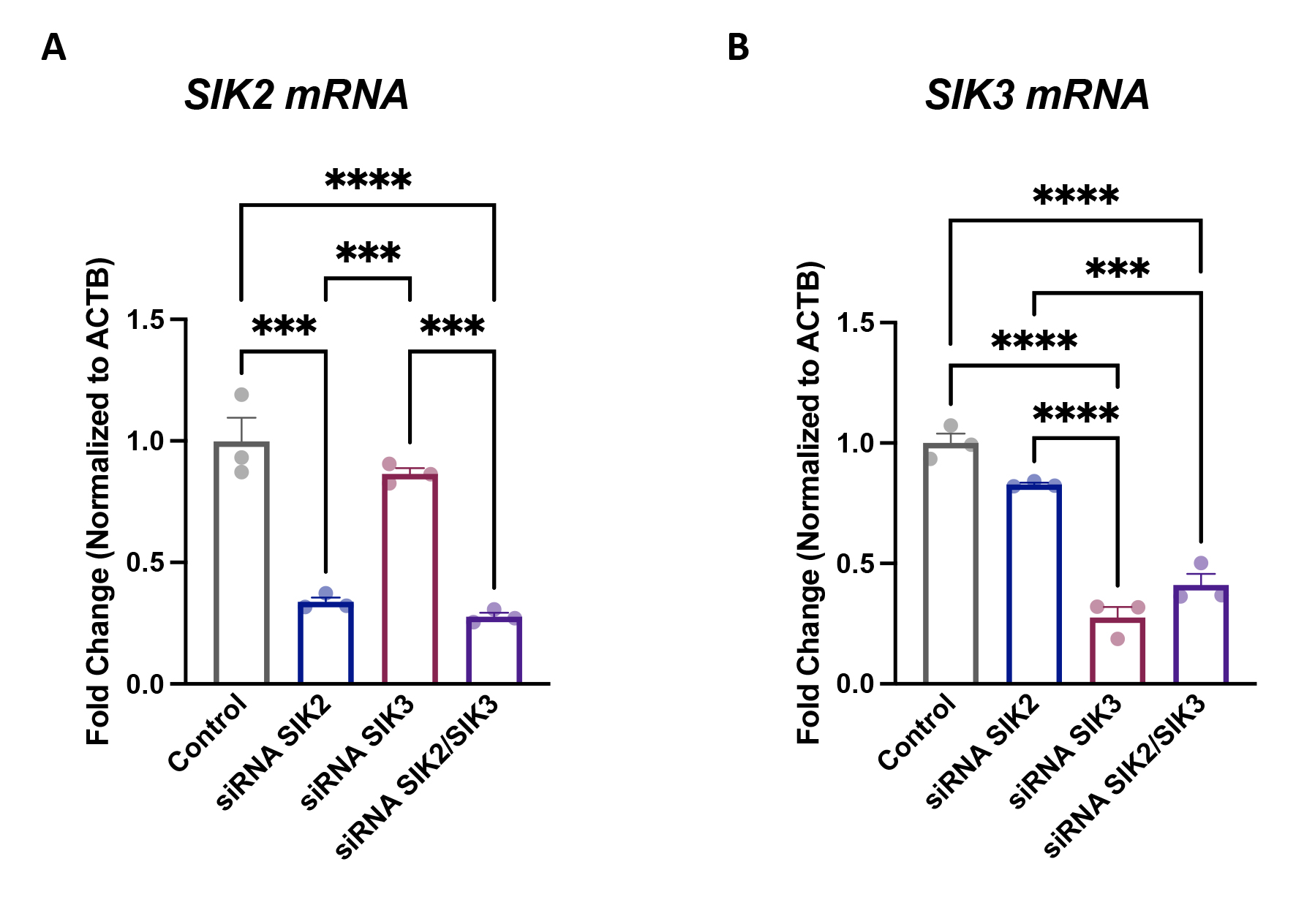

Supplement: Supplementary file 1 [file Image3.TIF]

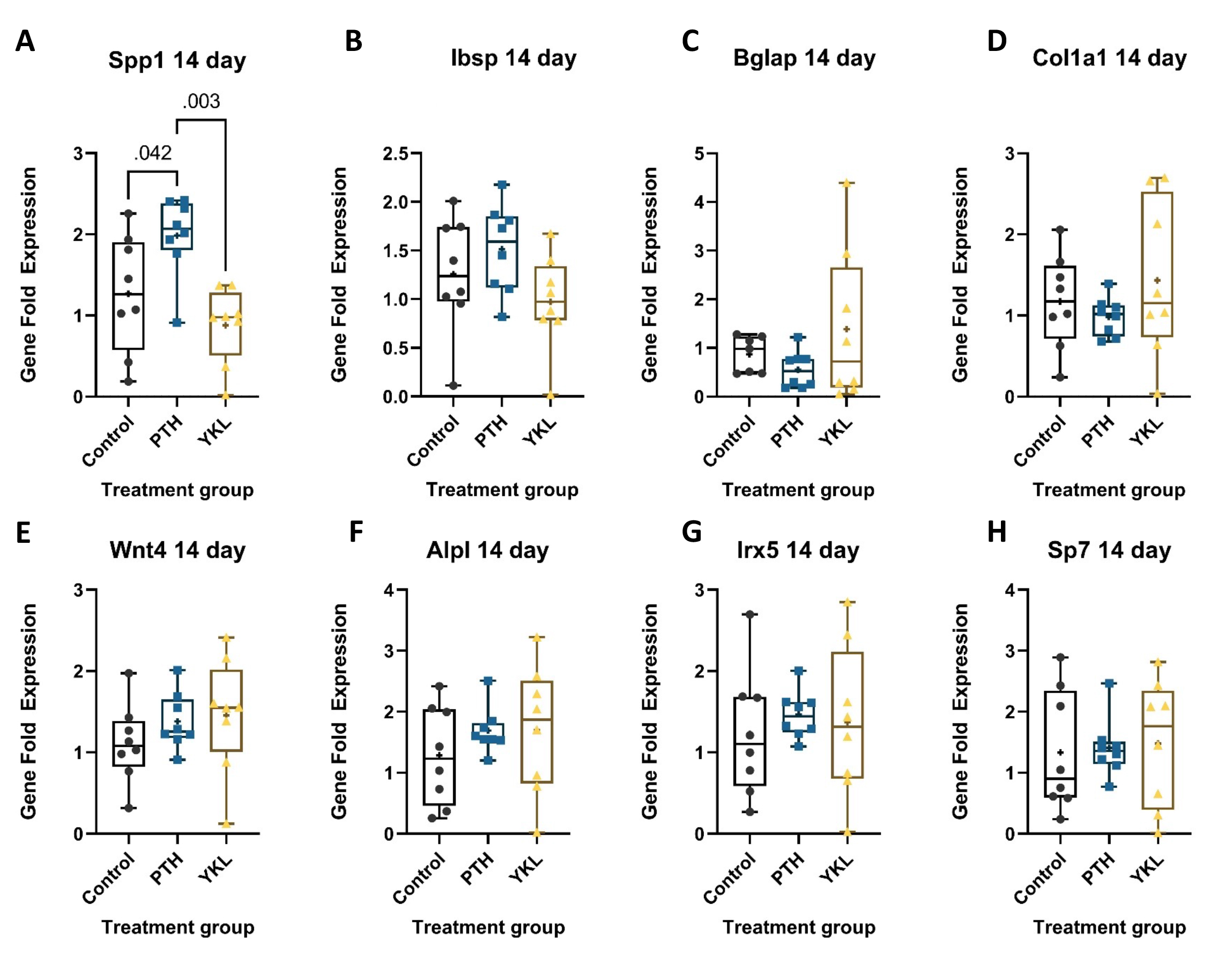

Supplement: Supplementary file 2 [file Image2.TIF]

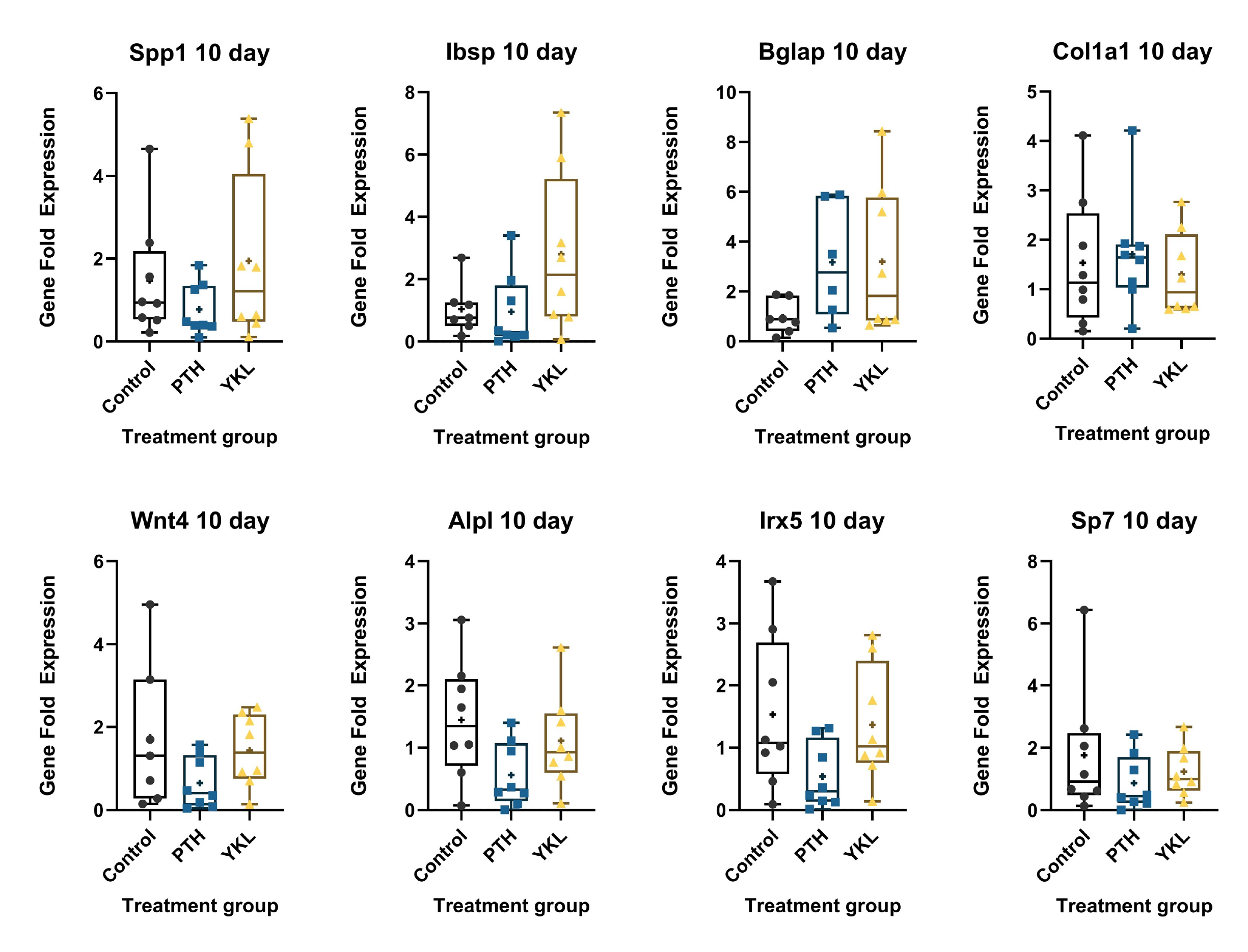

Supplement: Supplementary file 3 [file Image1.TIF]
